# Conserved role of Ras-GEFs in promoting aging: from yeast to mice

**DOI:** 10.18632/aging.100320

**Published:** 2011-04-26

**Authors:** Mario G. Mirisola, Valter D. Longo

**Affiliations:** ^1^ Dipartimento di Biotecnologie Mediche e forensi, via Divisi 83, Universita' di Palermo 90133 Palermo, Italy; ^2^ Andrus Gerontology Center, University of Southern California, 90089 Los Angeles, CA, USA

In a new article published in the journal Aging, Borras et al. report that Ras-GRF1 homozigous deletion increases both median and maximum longevity in laboratory mice [1]. The human Ras, superfamily now counts more than 150 different proteins subdivided into five different protein families: Ras, Rho, Rab, Arf and Ran. These protein families regulate many cellular processes [2] including cellular differentiation and proliferation (Ras), cell citoskeleton organization and cell shape (Rho), intracellular protein trafficking (Rab, Arf) and nucleo-citoplasmic transport (Ran).

Ras protein, the founding member of the small GTP binding protein superfamily, is found mutated in 30% of all human tumors and, in mammals, consists of three genes and four gene products (N-Ras, H-Ras, K-Ras4A and KRas4B). Many of these proteins are ubiquitously expressed but regulated by a multitude of specific Guanine nucleotide Exchange Factors (GEFs) and GTPase Activating Proteins (GAPs). In fact, even though this protein superfamily has the endogenous capability to hydrolyze the bound GTP, GEFs and GAPs, respectively catalyze the activating and the inactivating reactions [3]. It is interesting to note that, although Ras-GRF1 (one of the mammalian GEFs) shows only partial homology to the yeast CDC25 (one of the two yeast GEFs) and mammalian and yeast Ras proteins have limited functional homology, both exchange factors are regulated by the PKA serine/threonine kinase [4, 5] suggesting the existence of conserved Ras-dependent signaling networks. Both RasGRFs were first discovered for their ability to exchange the nucleotide bound to Ras proteins [6, 7] but these multidomain proteins can have additional activities.

Other than the REM and CDC25 domain, capable of exchanging the Ras-bound GDP, full length RasGRFs contain, in fact, a PH domain that can interact with the NGF receptor TrkA [31] and an IQ domain capable of calmodulin binding and responsible for calcium modulation [8; 9]. It also contains a second PH-DH domain that is capable of binding to membrane bound PI[4, 5]P2, microtubules [10], phosphatidic acid, Rho and Rac GTPase [11, 12, 5] and spinophilin, a scaffold protein that interact with actin filaments and p70 S6 kinase [13]. It is therefore possible that the RasGRF1 −/− mouse phenotype may be due to the impairment of GTP binding proteins other than Ras or to inhibition of other signaling cascades. However, it must be noted that Ras-GRF1 signaling is required for normal beta cell development and glucose homeostasis and that isolated islet from GRF1 knockout fail to activate Akt and Erk [14] suggesting a major role of RasGRF1 in Ras activation in this cell type. A clear but much reduced effect of the same knockout can be also seen in the amount of activated Erk protein in isolated retina [15].

Ras-GRF1 was previously implicated in beta cell langherans islet development, glucose homeostasis [14], learning and memory impairment and retinal defects [16, 17, 18]. More recently [19] Ras-GRF1 has been invoked as a possible explanation for the longevity observed in mice obtained without paternal contribution [20]. These mice generated using two sets of female genomes display increased average longevity and reduced body weight. Since the RasGRF1 locus is imprinted in female gametogenesis, leaving the whole protein production to the paternal allele, it was argued that bi-maternal condition is functionally equivalent to the RasGRF1 deletion [19].

Ras-GRF1 is normally expressed in brain (hypothalamus and hippocampus), pancreatic cells and skeletal muscle [21]. Messenger length and composition are quite heterogeneous because the Ras-GRF1 locus is heavily affected by alternative splicing. This results in a variety of mRNA isoforms that show tissue and developmental specific expression. Consequently, protein isoforms range from a 140 KDa full-length protein expressed in brain and in pancreatic islet to the smallest 20kDa isoform Ras-GRFβ, expressed in mouse pancreas [21]. These isoforms share only some of the functional domains raising the possibility that different isoforms may perform different tasks.

In their study, Borras et al. [1] find increased average and maximal Lifespan in *RasGrf1−/−* mice. Survival curves revealed a marked increase (20%) in the average lifespan of *RasGrf1−/−* male mice (mean values WT: 100.5±4.2 weeks and RasGrf1−/−: 120.7±4.7 weeks; median WT: 104 weeks; RasGrf1−/−: 124 weeks) and mice with *RasGrf1−/−* showed better motor coordination than controls. At the molecular level they find: a) Increased Expression of the 16S rRNA in *RasGrf1−/−* mice. 16S rRNA is one of the mitochondrial rRNA and mitochondrial gene expression and function has been demonstrated to positively correlate with longevity in organisms from yeast to human [40, 41, 42, 43] b) Increased expression of SIRT1 in *RasGrf1−/−* mice. Sirtuins play a role in a variety of diseases [44], but their importance in mammalian lifespan is not clear [45]. c) Maintained in vivo glucose uptake in aged *RasGrf1−/−* mice.

Reduced glucose uptake is associated with aging [39]. They analyzed the in vivo glucose uptake in young and old animals showing higher uptake levels in *RasGRF1−/−* mice with respect to the wild type of the same age, d) RasGRF1−/− mice displayed reduced IGF1 activity and blood metabolomic analysis showed clear similarities with calorie-restricted animals. IGF1 activity has a consistent effect on the aging process [23], suggesting that the downstream Ras pathway may play a central role in this process in mammals as it does in yeast [23]. Higher glycogen content in mice depleted of the RasGRF1 was also observed. Notably, glycogen and trehalose content positively correlates with stress resistance and Ras/AC/PKA depletion in yeast while constitutively activated Ras/AC/PKA pathway reduces both carbohydrate content [22]. Borras et al. suggest the longevity phenotype observed in mice with homozygous Ras-GRF1 deletion may be the outcome of decreased Ras activity.

In yeast the Ras, Tor and Sch9 signaling pathways are partly responsible for integrating nutrient inputs into cell growth, division and aging [23]. Impairment of the Ras/AC/PKA pathway increases stress resistance and longevity [24, 25, 26] through different downstream effectors whose roles are functionally conserved in higher eukaryotes including mammals [23]. Mechanistically, downregulation of Ras/AC/PKA in yeast provokes relocalization/activation of the transcription factors Msn2/4 [28], activation of the transcription factors Gis1 through Rim15, [29, 26, 28, 30] and Hsf1 activation [29]. All these factors enhance cellular protection systems activating stress response genes such as SODs, catalase, HSPs, autophagy and probably many others. [31, 23, 27, 28].

Orthologs of genes that function downstream of Ras in yeast have also been implicated in longevity extension in mice. Deletion of the mouse adenylate cyclase type 5 and the consequently PKA downregulation extends lifespan, stress resistance and mediates upregulation of SODs [32] and mice lacking PKA RII or Cβ subunits are protected from age-related deleterious changes such as weight gain, hypertrophic liver and cardiac dysfunction and enlargement [33, 34]. In addition, serum from a cohort of individuals with Growth Hormone Receptor (GHR) mutations, showing very low cancer and diabetes incidence, inhibited the expression of N-Ras, TOR and PKA when added to cultured primary human epithelial cells [35].

It is therefore tempting to propose that the pro-longevity effect of the homozygous Ras-GRF1 deletion observed by Borras is due to a reduced activity of the Ras or an analogous pathway and to consider that the increased longevity observed may be evidence for the conserved role of the Ras pathway in the aging process in organisms ranging from yeast to mammals [49]. However, the presence of more isoforms of the Ras-GRF1 coupled with the loose ligand specificity of Ras-GRF1 that is capable to bind Ras, Rac, Rho GTPase, microtubules, PI[4, 5]P2 and fosfatidic acid indicates that additional studies are necessary to determine how altered Ras-GRF1 signaling promotes aging in mammals.

In summary, the identification of a pro-aging role of Ras in aging therapy is an important and welcomed discovery especially considering that drug companies have identified a number of compounds capable to inhibit GEFs such as Brefeldin A (large ArfGef), SecinH3 (small ArfGef) and NSC23766 (RacGef) [36, 37, 38]. These examples demonstrate that pharmacological GEF inhibition may be a feasible strategy to extend longevity. In particular, the possibility to obtain GEF inhibition, stabilizing the interaction between the G protein and its GEF, an approach described as “interfacial inhibition” [36], is very interesting because the compound doesn't need to compete with the natural substrate and hence inhibition may be obtained even by low affinity compounds. Other interfacial inhibitors are already in clinical practice. Rapamycin for example, which inhibits mTOR signaling, has been shown to extend the life span of mice [46, 47, 48].

## References

[R1] Borrás C, Monleón D, López-Grueso R, Gambini J, Orlando L, Pallardó FV, Santos E, Viña J, Font de Mora J (2011). RasGrf1 deficiency delays aging in mice. Aging.

[R2] Bos JL, Rehmann H, Wittinghofer A (2007). GEFs and GAPs: critical elements in the control of small G proteins. Cell.

[R3] Wittinghofer A, Nassar N (1996). How Ras-related proteins talk to their effectors. Trends Biochem Sci.

[R4] Gross E, Goldberg D, Levitzki A (1992). Phosphorylation of the S. cerevisiae Cdc25 in response to glucose results in its dissociation from Ras. Nature.

[R5] Buday L, Downward J (2008). Many faces of Ras activation. Biochim Biophys Acta.

[R6] Shou C, Farnsworth CL, Neel BG, Feig LA (1992). Molecular cloning of cDNAs encoding a guanine-nucleotide-releasing factor for Ras p21. Nature.

[R7] Martegani E, Vanoni M, Zippel R, Coccetti P, Brambilla R, Ferrari C, Sturani E, Alberghina L (1992). Cloning by functional complementation of a mouse cDNA encoding a homologue of CDC25, a Saccharomyces cerevisiae RAS activator. EMBO J.

[R8] Farnsworth CL, Freshney NW, Rosen LB, Ghosh A, Greenberg ME, Feig LA (1995). Calcium activation of Ras mediated by neuronal exchange factor Ras-GRF. Nature.

[R9] Agell N, Bachs O, Rocamora N, Villalonga P (2002). Modulation of the Ras/Raf/MEK/ERK pathway by Ca(2+), and calmodulin. Cell Signal.

[R10] Forlani G, Baldassa S, Lavagni P, Sturani E, Zippel R (2006). The guanine nucleotide exchange factor RasGRF1 directly binds microtubules via DHPH2-mediated interaction. FEBS J.

[R11] Cen H, Papageorge AG, Zippel R, Lowy DR, Zhang K (1992). Isolation of multiple mouse cDNAs with coding homology to Saccharomyces cerevisiae CDC25: identification of a region related to Bcr, Vav, Dbl and CDC24. EMBO J.

[R12] Tung PS, Fam NP, Chen L, Moran MF (1997). A 54-kDa protein related to ras-guanine nucleotide release factor expressed in the rat exocrine pancreas. Cell Tissue Res.

[R13] Buchsbaum RJ, Connolly BA, Feig LA (2003). Regulation of p70 S6 kinase by complex formation between the Rac guanine nucleotide exchange factor (Rac-GEF) Tiam1 and the scaffold spinophilin. J Biol Chem.

[R14] Font de Mora J, Esteban LM, Burks DJ, Núñez A, Garcés C, García-Barrado MJ, Iglesias-Osma MC, Moratinos J, Ward JM, Santos E (2003). Ras-GRF1 signaling is required for normal beta-cell development and glucose homeostasis. EMBO J.

[R15] Fernández-Medarde A, Barhoum R, Riquelme R, Porteros A, Núñez A, de Luis A, de Las Rivas J, de la Villa P, Varela-Nieto I, Santos E (2009). RasGRF1 disruption causes retinal photoreception defects and associated transcriptomic alterations. J Neurochem.

[R16] Brambilla R, Gnesutta N, Minichiello L, White G, Roylance AJ, Herron CE, Ramsey M, Wolfer DP, Cestari V, Rossi-Arnaud C, Grant SG, Chapman PF, Lipp HP, Sturani E, Klein R (1997). A role for the Ras signalling pathway in synaptic transmission and long-term memory. Nature.

[R17] Giese KP, Friedman E, Telliez JB, Fedorov NB, Wines M, Feig LA, Silva AJ (2001). Hippocampus-dependent learning and memory is impaired in mice lacking the Ras-guanine-nucleotide releasing factor 1 (Ras-GRF1). Neuropharmacology.

[R18] Li S, Tian X, Hartley DM, Feig LA (2006). The environment versus genetics in controlling the contribution of MAP kinases to synaptic plasticity. Curr Biol.

[R19] de Magalhães JP (2011). Paternal genome effects on aging: evidence for a role of Rasgrf1 in longevity determination?. Mech Ageing Dev.

[R20] Kawahara M, Kono T (2010). Longevity in mice without a father. Hum Reprod.

[R21] Fernández-Medarde A, Santos E (2011). The RasGrf family of mammalian guanine nucleotide exchange factors. Biochim Biophys Acta.

[R22] Geymonat M, Wang L, Garreau H, Jacquet M (1998). Ssa1p chaperone interacts with the guanine nucleotide exchange factor of ras Cdc25p and controls the cAMP pathway in Saccharomyces cerevisiae. Mol Microbiol.

[R23] Fontana L, Partridge L, Longo VD (2010). Extending healthy life span--from yeast to humans. Science.

[R24] Longo Valter D The Chronological lifespan of Saccharomyces cerevisiae: Studies of SOD, Ras and BclII.

[R25] Fabrizio P, Pozza F, Pletcher SD, Gendron CM, Longo VD (2001). Regulation of longevity and stress resistance by Sch9 in yeast. Science.

[R26] Wei M, Fabrizio P, Hu J, Ge H, Cheng C, Li L, Longo VD (2008). Life span extension by calorie restriction depends on Rim15 and transcription factors downstream of Ras/PKA, Tor, and Sch9. PLoS Genet.

[R27] Fabrizio P, Liou LL, Moy VN, Diaspro A, Valentine JS, Gralla EB, Longo VD (2003). SOD2 functions downstream of Sch9 to extend longevity in yeast. Genetics.

[R28] Martínez-Pastor MT, Marchler G, Schüller C, Marchler-Bauer A, Ruis H, Estruch F (1996). The Saccharomyces cerevisiae zinc finger proteins Msn2p and Msn4p are required for transcriptional induction through the stress response element (STRE). EMBO J.

[R29] Anderson ES, Harshaw RB, Thate T, Craig NL, Nelson HC (2005). Protein kinase A regulates constitutive expression of small heat-shock genes in an Msn2 /4p-independent and Hsf1p-dependent manner in Saccharomyces cerevisiae Ferguson SB. Genetics.

[R30] Pedruzzi I, Dubouloz F, Cameroni E, Wanke V, Roosen J, Winderickx J, De Virgilio C (2003). TOR and PKA signaling pathways converge on the protein kinase Rim15 to control entry into G0. Mol Cell.

[R31] MacDonald JI, Verdi JM, Meakin SO (1999). Activity-dependent interaction of the intracellular domain of rat trkA with intermediate filament proteins, the beta-6 proteasomal subunit, Ras-GRF1, and the p162 subunit of eIF3. J Mol Neurosci.

[R32] Yan L, Vatner DE, O'Connor JP, Ivessa A, Ge H, Chen W, Hirotani S, Ishikawa Y, Sadoshima J, Vatner SF (2007). Type 5 adenylyl cyclase disruption increases longevity and protects against stress. Cell.

[R33] Enns LC, Morton JF, Treuting PR, Emond MJ, Wolf NS, Dai DF, McKnight GS, Rabinovitch PS, Ladiges WC (2009). Disruption of protein kinase A in mice enhances healthy aging. PLoS One.

[R34] Enns LC, Pettan-Brewer C, Ladiges W (2010). Protein kinase A is a target for aging and the aging heart. Aging (Albany NY).

[R35] Guevara-Aguirre J, Balasubramanian P, Guevara-Aguirre M, Wei M, Madia F, Cheng CW, Hwang D, Martin-Montalvo A, Saavedra J, Ingles S, de Cabo R, Cohen P, Longo VD (2011). Growth hormone receptor deficiency is associated with a major reduction in pro-aging signaling, cancer, and diabetes in humans. Sci Transl Med.

[R36] Renault L, Guibert B, Cherfils J (2003). Structural snapshots of the mechanism and inhibition of a guanine nucleotide exchange factor. Nature.

[R37] Hafner M, Schmitz A, Grüne I, Srivatsan SG, Paul B, Kolanus W, Quast T, Kremmer E, Bauer I, Famulok M (2006). Inhibition of cytohesins by SecinH3 leads to hepatic insulin resistance. Nature.

[R38] Gao Y, Dickerson JB, Guo F, Zheng J, Zheng Y (2004). Rational design and characterization of a Rac GTPase-specific small molecule inhibitor. Proc Natl Acad Sci U S A.

[R39] Borras C, Stvolinsky S, Lopez-Grueso R, Fedorova T, Gambini J, Boldyrev A, Vina J (2009). Low in vivo brain glucose consumption and high oxidative stress in accelerated aging. FEBS Lett.

[R40] Guo Z, Adomas AB, Jackson ED, Qin H, Townsend JP (2011). SIR2 and other genes are abundantly expressed in long-lived natural segregants for replicative aging of the budding yeast Saccharomyces cerevisiae. FEMS Yeast Res.

[R41] Zahn JM, Poosala S, Owen AB, Ingram DK, Lustig A, Carter A, Weeraratna AT, Taub DD, Gorospe M, Mazan-Mamczarz K, Lakatta EG, Boheler KR, Xu X, Mattson MP, Falco G, Ko MS, Schlessinger D, Firman J, Kummerfeld SK, Wood WH, Zonderman AB, Kim SK, Becker KG (2007). AGEMAP: a gene expression database for aging in mice. PLoS Genet.

[R42] Cho J, Hur JH, Walker DW (2011). The role of mitochondria in Drosophila aging. Exp Gerontol.

[R43] Borras C, Gambini J, Vina J (2007). Mitochondrial oxidant generation is involved in determining why females live longer than males. Front Biosci.

[R44] Guarente L (2006). Sirtuins as potential targets for metabolic syndrome. Nature.

[R45] Herranz D, Serrano M (2010). SIRT1: recent lessons from mouse models. Nat Rev Cancer.

[R46] Brown EJ, Albers MW, Shin TB, Ichikawa K, Keith CT, Lane WS, Schreiber SL (1994). A mammalian protein targeted by G1-arresting rapamycin-receptor complex. Nature.

[R47] Anisimov VN, Zabezhinski MA, Popovich IG, Piskunova TS, Semenchenko AV, Tyndyk ML, Yurova MN, Antoch MP, Blagosklonny MV (2010). Rapamycin extends maximal lifespan in cancer-prone mice. Am J Pathol.

[R48] Harrison DE, Strong R, Sharp ZD, Nelson JF, Astle CM, Flurkey K, Nadon NL, Wilkinson JE, Frenkel K, Carter CS, Pahor M, Javors MA, Fernandez E, Miller RA (2009). Rapamycin fed late in life extends lifespan in genetically heterogeneous mice. Nature.

[R49] Longo VD (1999). Mutations in signal transduction proteins increase stress resistance and longevity in yeast, nematodes, fruit flies, and mammalian neuronal cells. Neurobiol Aging.

